# Experimental Research on Quality Parameters of Recycled Concrete

**DOI:** 10.3390/ma13112538

**Published:** 2020-06-03

**Authors:** Ramunė Žurauskienė, Marina Valentukevičienė

**Affiliations:** 1 Department of Building Materials and Fire Safety, Faculty of Civil Engineering, Vilnius Gediminas Technical University, LT-10223 Vilnius, Lithuania; ramune.zurauskiene@vgtu.lt; 2 Department of Environmental Protection and Water Engineering, Faculty of Environmental Engineering, Vilnius Gediminas Technical University, LT-10223 Vilnius, Lithuania

**Keywords:** coarse aggregate, concrete waste, fresh concrete

## Abstract

Concrete itself, and issues relating to the recycling and management of reinforced concrete waste, are highly relevant, especially when urban expansion is being achieved by increased building construction volumes. This research investigates concrete waste and its (re)usage possibilities and resolves several major issues related to the question of how natural materials can be replaced by compounds made from concrete waste, thereby saving natural resources. The experiment was carried out using concrete mixtures, which were combined with natural aggregates and crushed concrete waste (fraction 4/16). The resulting mix of concrete was achieved using natural aggregates, thus replacing natural aggregates with waste, which had partially and fully replaced bulky aggregates with crushed concrete waste. The main aim of the investigation was to investigate how aggregates made from crushed concrete waste impact the properties of concrete. The exothermic effect on the concrete mixture during the hardening process was investigated. Furthermore, a macrostructural analysis of hardened concrete was conducted using scanned sample images; the adhesion zone between newly formed concrete stone and aggregates derived from natural rock from crushed concrete waste was investigated. Using an electron microscope to observe aggregate from crushed concrete waste and the contact zone of hardened cement stone revealed that the aggregate from waste adheres poorly with hardened cement stone. Furthermore, both the mechanical properties of new, hardened concrete and determined resistance to frost indicators are weak. Concrete density and compression strength decreased (by up to 8% and up to 18%, respectively), and absorption increased almost twofold due to aggregates derived from crushed concrete waste, since their cleavage strength indicator was twice as high, while water absorption was four times higher than that of natural aggregate. The results indicate that recycled concrete obtained from demolished buildings is environmentally sustainable and can be recommended for lower quality concrete for use in related engineering projects.

## 1. Introduction

The world is intensively attempting to solve concrete waste recycling and re-usage problems. Concrete waste can be used as an aggregate in newly mixed concrete production [1]. Many researchers, after doing research on the effect of concrete waste on hardened concrete properties, state that the waste worsens the properties of concrete building products, as in, concrete strength lowers while water absorption rises [2]. Currently, scientific research in the examined subject is directed towards appropriate addition and admixture research and how to rationally use concrete waste in new quality product production [3,4,5,6]. To develop concrete waste usage in fresh concrete, scientific research is a must, by choosing the most appropriate fresh concrete compositions or by researching additions without admixture, which would improve concrete physical and mechanical properties [7,8]. This way, concrete waste can be used in used new ecological concrete production technological cycles [9,10]. By not using additives and admixtures, while lower class, quality ecological concrete can be produced [11]. How the concrete properties change when using additives from crushed concrete waste has to be extensively researched [12,13]. Research has to encompass not only mechanical property changes but exploitation properties, like resistance to frost, acid attack, abrasion and leaching [14].

**Figure 1:**
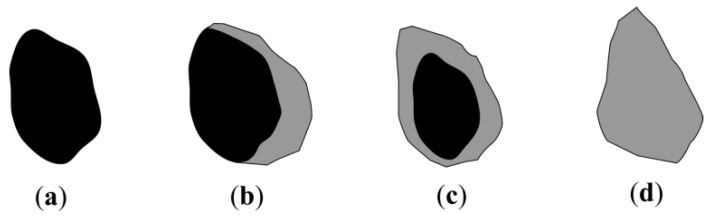
Types of breakstone prepared from crushed concrete waste: (**a**) Natural aggregate; (**b**) Natural aggregate partially covered with old concrete parts; (**c**) Natural aggregate fully covered with old concrete parts; (**d**) crushed old concrete part.

Coarse aggregate is produced by crushing and sieving concrete waste. This aggregate consists of several types of particles (Figure 1): natural aggregate, which splits off during the crushing process and does not have any old concrete particles attached to it (Figure 1a), natural aggregate with old concrete attached to it on one or several sides (Figure 1b,c), as well as particles consisting of only crushed old concrete (Figure 1d). Using earlier research, it was determined [15] that the obtained aggregate particles are oblong in shape and according to the particle form indicator, the obtained aggregate is labelled as the SI30 category [16]. 

In the presence of such composition concrete aggregates, fresh concrete is of two types, according to mechanical properties. Aggregates, that is to say, rock, is characterized by a high compression strength (100–250 MPa), and lower mechanical strength aggregates (45–65 MPa depending on past concrete mechanical strength). Due to this reason, some changes to fresh concrete parts can be seen when calculating concrete composition. In this fresh concrete, a higher amount of concrete is used than in concrete with aggregates from natural rocks. Additionally, a higher amount of water is used, since crushed concrete aggregates are characterized by a higher need for water, and to mix the mixture, a higher amount of concrete is used.

Environmental performance and environmental life cycle assessment (LCA) of the manufacture of reused concrete, along with uncertainty of shear resistance models are the best tools according to related research carried out by leading scientists [17,18,19,20] 

The goal of this work is to not only research concrete, which is produced from crushed old concrete waste instead of coarse aggregate, and mechanical properties, but also the exploitation properties and structural indicators. By using coarse aggregate from crushed concrete waste for research, it is possible to determine the hardened concrete class and mechanical properties of ecological concrete.

## 2. Materials and Methods

In this work, a binding material was used—Portland cement CEM II/A-LL 42.5 N—which corresponds to LST EN 197-1:2011/P:2013 standard requirements. For a fine aggregate, natural sand was used, which has a bulk density of 1.63 g/cm^3^, a particle density of 2.43 g/cm^3^, and the particle size is from 0.125 up to 4 mm. This aggregate fits LST EN 12620:2003+A1:2008 standard requirements.

Two types of coarse aggregates were used: natural gravel and crushed concrete waste. Crushed concrete waste aggregate was prepared from concrete waste brought from three different crushing sites. This concrete waste was not contaminated with any salts or chemicals since it was picked out in the building material waste site by hand. When wanting to use aggregates made from concrete waste in concrete production, it is important that they are close to the compression strength class of the produced concrete. Due to that, the crushed constructions have to be picked out and sorted beforehand. During raw material research, the strength of crushed concrete constructions was determined. Cubes of 100 mm × 100 mm × 100 mm were cut from brought waste and their compression strength was determined (from 45 to 65 MPa). Crushed concrete pieces were sieved through 16 mm and 4 mm meshes. The main characteristics of this aggregate are shown in Table 1. For this work, natural gravel was also used, and its main properties are shown in Table 1.

All concrete compositions were picked according to raw material characteristics with the calculating experimental method by using the computer program “AGbetonas” (2003). This program was created using concrete standard recommendations. The mixed fresh concrete slump is 3 cm.

In this work, research was carried out by changing the amount of concrete waste and all coarse aggregate amounts in concrete. Due to this, three fresh concrete mixtures were prepared, which were marked CW0, CW1, and CW2. Fresh concrete compositions are presented in Table 2. In the research, coarse aggregates were used which had fractions of 4/16. In the CW0 mixture, the coarse aggregate was crushed gravel, CW1 (crushed gravel and crushed concrete waste mixture), and CW2 (only crushed concrete waste). The w/c ratio was kept the same so that different water amounts would not change the concrete mixture properties at the starting period and would not influence the hardened concrete properties. First of all, the fresh concrete mixture composition CW0 was calculated by using a concrete property calculation program, which is based on the volume method and adjusted for used material properties. Afterwards, without changing composite material amounts, natural gravel was replaced by crushed concrete waste. In the mixture CW1, less than 50% of the gravel was replaced with waste in the belief that the obtained concrete properties would not change significantly.

According to the provided concrete compositions, 100 mm × 100 mm × 100 mm concrete cubes were formed. Their hardening was carried out according to EN 206-1 standard requirements.

Exothermic process temperatures in the fresh concrete mixture were determined by a methodology created by the “Alcoa” firm (Pittsburgh, PA, USA). A fresh concrete sample weighing 1.5 kg was placed into 100 mm × 100 mm × 100 mm textolite vessels. After filling the vessel with mixture, the sample had a T-type thermocouple placed in it, which was then placed into a glass tube. The thermocouple was connected to a data-transmitting device and a personal computer. Instantly after the fresh concrete was mixed, the form was placed into a metal box and isolated with 50 mm thick polystyrene foam. Temperature rises done in an automatic method were constantly recorded into the computer without any interruptions.

The concrete samples’ main properties were determined while upholding methodologies specified in normative documents: concrete sample density according to LST EN 12390-7:2019, concrete sample absorption according to LST EN 13369:2018, and sample compression strength according to LST EN 12390-3:2019. Samples were compressed with an “AUTOMAX 3000” (Herzele, Belgium) press, corresponding to LST EN 12390-4:2019.

Using the methodology in [24,25], concrete sample structural indicators were determined: structural unevenness indicator N, capillary mass stream speed in normal conditions according to the direction of freezing g_2_, effective porosity W_E_, general active porosity W_R_, porous space reserve R, capillary mass stream speed in a vacuum perpendicular to freezing temperatures G_1_, and capillary mass stream speed in a vacuum in the direction of freezing G_2_. The sample predicted exploitation resistance to frost was calculated according to the sample structural characteristics, using predictive formulas according to [26]. 

The sample destruction start can be calculated using this formula:

(1) $$F=0.231\frac{R^{1.068}\cdot D^{1.345}\cdot G_{1}^{0.275}\cdot G_{2}^{0.663}}{N^{0.285}\cdot g_{1}^{0.830}}$$

where *R*—porous space reserve,%; *D*—conditional pore and capillary wall strength; *G*_1_—capillary mass stream speed in a vacuum in the direction of freezing; *G*_2_—capillary mass stream speed perpendicular to freezing direction; *N*—structural direction unevenness indicator; g_1_—capillary mass stream speed during normal conditions.

The scanned sample image was investigated with a video creation program. The image was scanned with 800 dpi image quality. For image creation, the Adobe Photoshop CS2 program was used, using different colours to illustrate different phases: pores, hardened cement rock together with fine aggregate, coarse aggregate from natural rock, and coarse aggregate from crushed concrete waste.

**Figure 2:**
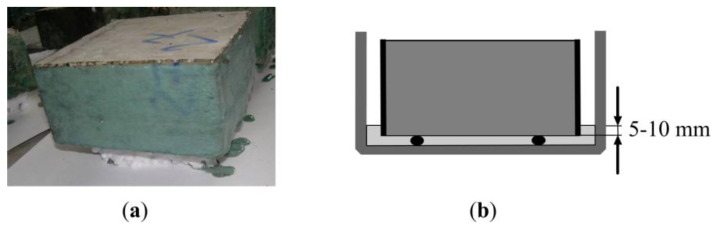
Sample view during frost resistance test: (**a**) view of the sample reinforced with polyester resin and fibre; (**b**) test scheme.

**Figure 3:**
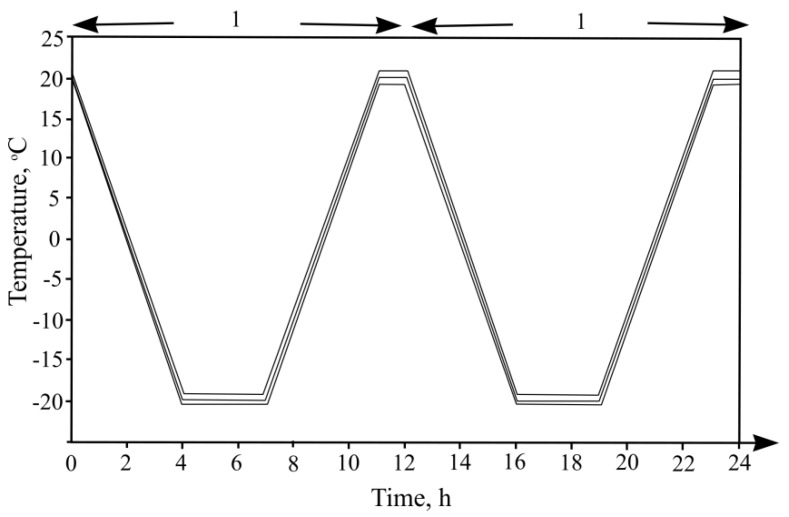
Temperature variation curve.

The sample resistance to frost was indicated according to technical conditions CEN/TS 12390-9:2006/P:2007. The experiment was carried out according to an alternative experiment method CF/CDF (without and with de-icing salts). Prepared concrete samples (Figure 2a) were weighed and added into vessels from two plates, so that the entire sample surface was submerged in the liquid (Figure 2b). The vessels with prepared samples were filled with water, and for 7 days, concrete samples were soaked in water at 20 ± 2 °C. After 7 days, the vessels were filled with 3% NaCl saline solution. After that, the vessels were placed into a chamber in which the samples were affected with a temperature fluctuation mode (Figure 3). After the experiment, the samples were dried and their mass loss was determined. If these losses were bigger than 1.5 kg/m^2^ (15 g/dm^2^), then the experiment ended.

**Figure 4:**
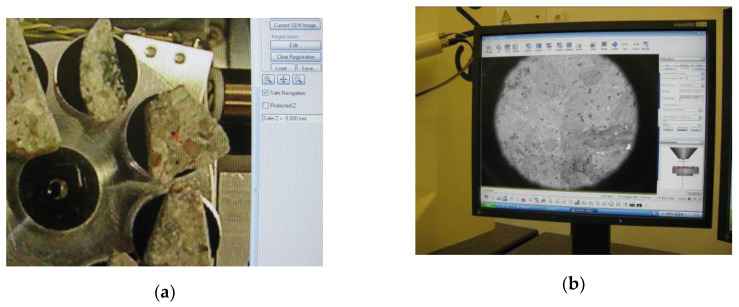
View of SEM search system: (**a**) real view of samples: (**b**) view of sample placed in the microscope.

The contact zone between the aggregate from concrete waste and newly formed cement rock was investigated with a scanning electron microscope, SEM EVO LS 25, Zeiss Germany (Oberkochen, Germany), using the navigational system (Figure 4). The image of concrete samples embedded on the microscope table view was put into the microscope image processing program (Figure 4a) and was paired with the current image in the microscope (Figure 4b).

The dominant space was marked in the program, which could be located in any part of the sample. Then the microscope lens/diaphragm pushed into the marked spot. After finding the contact zone, the image was zoomed in as necessary, so that it was be possible to see and fixate the dominant space.

## 3. Results and Discussions

**Figure 5:**
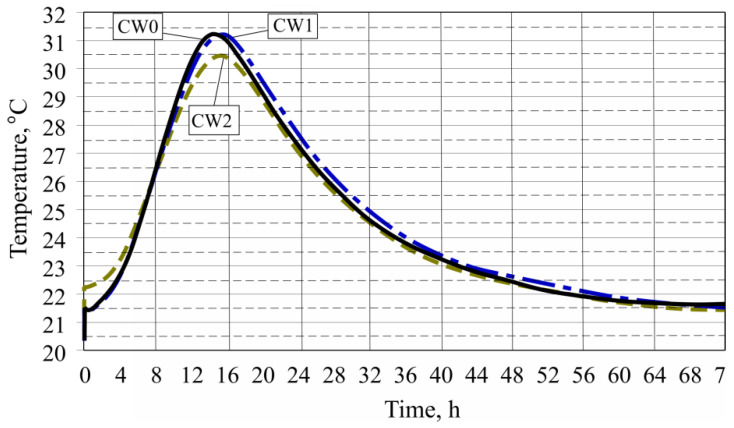
Dependence of the variation of exothermic effect temperatures on the amount of concrete waste during the hardening process.

To determine the aggregates, obtained from concrete crushing waste, the influence on fresh concrete hardening and the exothermic effect change during the Portland cement hydration duration was determined. Three fresh concrete samples have their compositions shown in Table 2 and exothermic effects shown in Figure 5.

By replacing part of the coarse aggregates with concrete waste, it can be noted that concrete waste insignificantly affects the mixture temperature T_max_, since both mixture temperatures’ highest value was almost the same. Fresh concrete, marked as CW0, and only has natural coarse aggregates, had a T_max_ of 31.28 °C, which was reached after 14 h 28 min. Meanwhile, after replacing part of the natural coarse aggregates with concrete waste (fresh concrete, which is marked CW1), a T_max_ of 31.24 °C was reached after 14 h 59 min. Mixture CW2 (15 h 34 min), which has coarse aggregates obtained by crushing concrete waste, had a T_max_ of 30.50 °C, while its reaching period lengthened by 1 h 6 min when compared with fresh concrete without waste’s (CW0—14 h 28 min) temperature, for which only natural coarse aggregates were used. Table 3 shows the fresh concrete hardening duration exothermic process indicator results.

Hardened concrete, made from researched mixture macrostructure analysis, was done by analyzing the sample cross-cut area and calculating each composite phase areas. In Table 2, CW0, CW1, and CW2 marked concrete cubes (100 mm × 100 mm × 100 mm), and after 28 days’ hardening, were cut and their visual composition component comparison was carried out.

**Figure 6:**
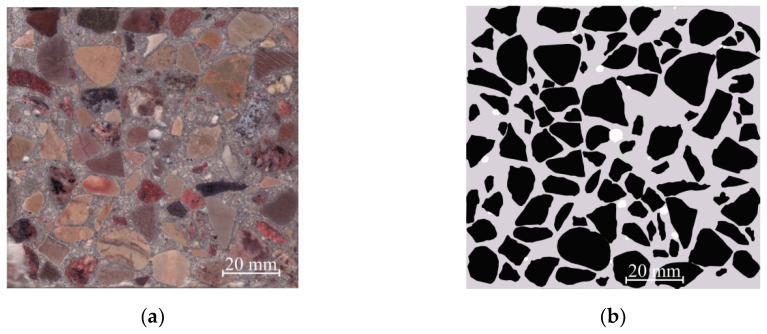
View of the concrete sample CW0, (**a**) scanned; (**b**) analyzed by employing computer graphic software: a white color is used to indicate pores; light gray—newly created cement stone with fine aggregate; black—coarse aggregates.

In Figure 6a, it can be seen that the coarse concrete aggregates are spread across the CW0 sample. Scanned image analysis data of the CW0 sample can be seen in Figure 6b. This picture shows that the cut has only pores, newly formed cement rock, and natural aggregates.

After calculating each colored zone in Figure 6b, it was determined that the white colored pores take up 0.64%, the light gray colored cement rock with fine aggregates—44.83%, and the black colored coarse aggregate—54.54%. Following the obtained area (Figure 6b) phase spread on the surface comparison with phase volumes in the whole concrete sample, it can be determined that coarse aggregates take up about 62% of the whole concrete volume.

**Figure 7:**
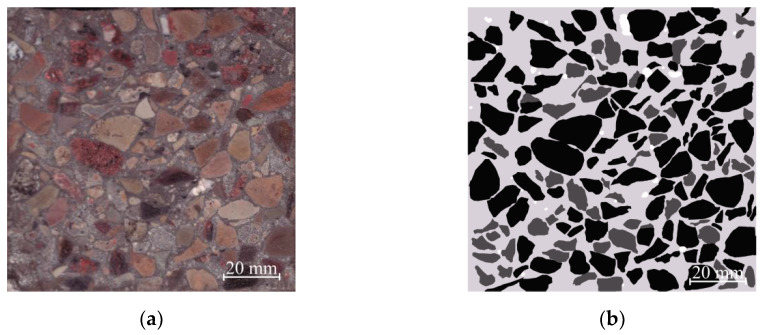
View of the concrete sample CW1, (**a**) scanned; (**b**) analyzed by employing computer graphic software: white color is used to indicate pores; light gray—newly created cement stone with fine aggregate; dark gray and black—concrete waste (dark gray—cement stone with fine aggregates and black—coarse aggregates).

Sample CW1 crosscut photos are shown in Figure 7a. This picture shows (differently from Figure 6) that a new component appears, which in Figure 7b is coloured in a dark gray color—aggregate from crushed concrete waste.

After calculating the different color areas, it was determined that white colored pores take up 0.81% (marked and included in calculations were only pores that had a diameter of ≥ 0.9 mm), light gray colored cement rock takes up 45.21%, and dark gray and black colored coarse aggregates from natural rock and crushed concrete waste—53.98% (black colored marked coarse natural aggregates take up 41.83%, while dark gray—that is, crushed old cement rock with fine aggregates which entered from crushed concrete waste—12.15%).

**Figure 8:**
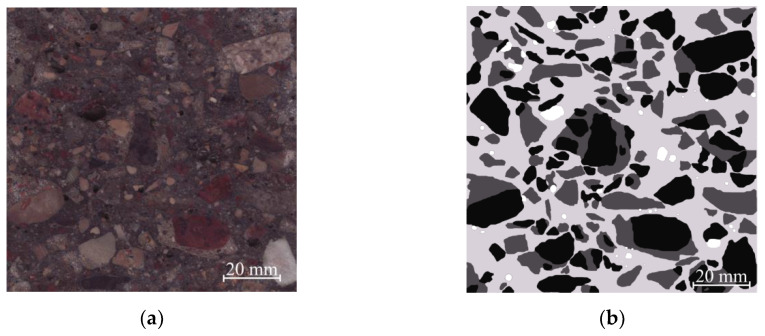
View of concrete sample CW2, (**a**) scanned; (**b**) analyzed by employing computer graphic software: white colored is used to indicate pores; light gray—newly created cement stone with fine aggregate; dark gray and black—concrete waste (dark gray—cement stone with fine aggregates and black—coarse aggregates).

In Figure 8a it can be seen how crushed concrete waste is spread across the sample marked as CW2. Its scanned image can be seen in Figure 8b.

Visually, it was noticed that half of the coarse aggregates during the crushing process are separated from the cement rock, while half the aggregates in the mixture are strongly stuck to the cement rock. Coarse aggregates in concrete are evenly spread through and they are all separated from one another with a mixed cement paste with fine aggregates. After calculating every colored zone area (Figure 8b) it was determined that white colored pores take up 1.43%, light gray colored cement rock—46.63%, and dark gray colored and black colored coarse aggregates from crushed concrete waste—50.95% (black colored coarse aggregates take up 27.07%, while dark gray—it is old crushed cement rock with fine aggregates—23.88%). By comparing the obtained area phase layout on the surface with phase volumes in the whole concrete sample, it can be determined that coarse aggregates take up 49% of the volume. This shows that coarse aggregates from crushed concrete waste in the concrete samples evenly spread throughout the entire area. Table 4 shows the concrete sample cross-cuts’ noticed component areas.

After completing the concrete made by using concrete waste macrostructure analysis, that is, after completing the concrete sample cut research and calculating every composite phase occupied area, it can be stated that the mixtures with waste (instead of natural coarse aggregate), evenly spread across the entire concrete mass. However, they encounter several problems: when mixing mixtures, air partially included into the fresh concrete, together with old concrete pieces, stay in there during compaction. Aggregates with cracks and fissures enter into the mixture. They are particles that appear during the crushing process when the hardened concrete strength, absorption, and other properties are negatively affected.

While analyzing concrete microstructure, examinations of microscopic contact zones were done, which allowed to determine the closeness connection between the cement rock and aggregate. With SEM, concrete samples were investigated after hardening for 28 days (CW0, CW2).

**Figure 9:**
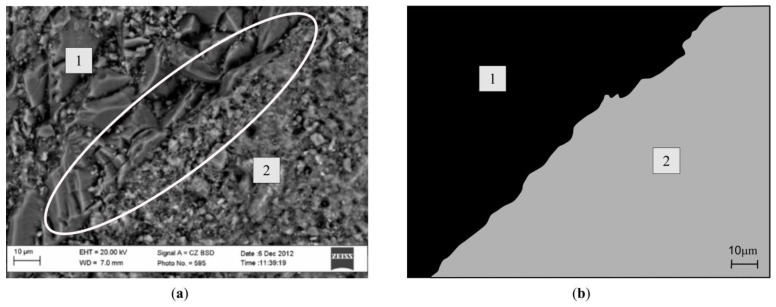
Contact area of the hardened cement stone and natural aggregate, (**a**) microscopic view; (**b**) view analyzed with computer graphic software; 1—natural aggregate; 2—cement stone.

Figure 9 shows the CW0 sample microscopic image. After investigating the natural aggregate and newly formed cement rock contact zone, it can be seen that the natural aggregate is finely sticking to newly formed cement rock. Between the natural aggregate and cement rock there are no cracks.

**Figure 10:**
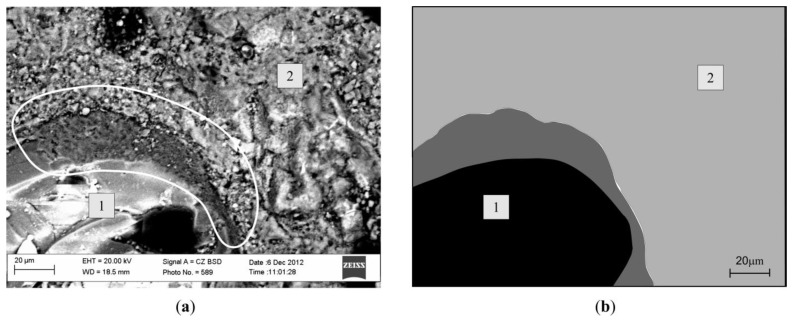
Contact area of new cement stone and concrete waste aggregate, (**a**) microscopic view; (**b**) view analyzed with computer graphic software; 1—aggregate obtained by crushing concrete waste; 2—new cement stone.

Figure 10 shows the aggregate from crushed concrete waste and hardened newly formed cement rock contact zone image (CW2). While carrying out microscopic research, it was noticed that aggregates from waste have weaker bonds with cement rock, and their contact zone has micro-cracks, which lowered the bond between the component parts. In Figure 10, it can be seen that between the aggregate and cement rock, there is a crack which separates the main concrete components. This shows the weakening of the component part bond.

Microscopic research showed that the contact zone between the waste and newly formed cement rock depends on the aggregate origin. When using natural aggregates, it can be noted that they bond well with hardened newly formed cement rock. When fresh concrete uses aggregates from waste, then between it and the newly formed cement rock a crack appears, separating these main fresh concrete components, and due to this, the contact between them weakens. To avoid the weakening of these zones, it is necessary to mix in an admixture and an addition to fresh concrete [27].

**Figure 11:**
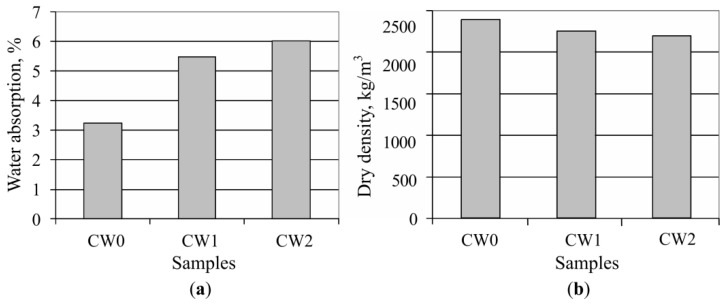
Concrete sample properties, (**a**) sample absorption; (**b**) sample density.

The hardened concrete sample absorption after three days of submersion in water was determined by researching the physical and mechanical properties. The results are shown in Figure 11a. It is seen that the highest sample absorption was obtained by submerging CW2 samples (6.02%), while the lowest was the CW0 sample—3.24%. The dried concrete sample volume is shown in Figure 11b. The concrete sample CW1, which has part of the coarse aggregates replaced with waste, the volume fell by about 5% when compared with sample CW0. By replacing all coarse aggregate amounts with waste concrete, sample CW2’s volume fell by about 8%. However, all the concrete sample volumes satisfy normal weight concrete requirements for volume: 2000–2600 kg/m^3^ [28].

**Figure 12:**
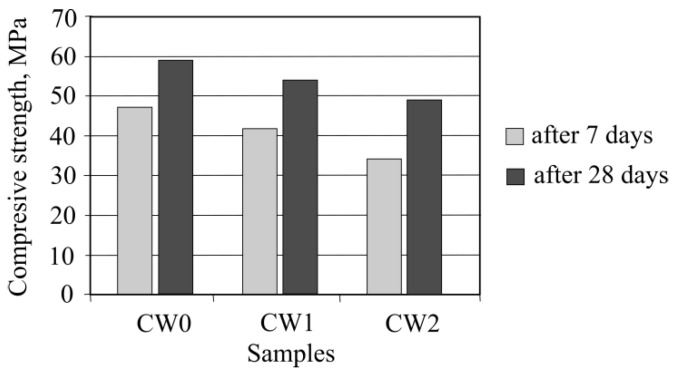
Dependence of samples’ compressive strength on the amount of concrete waste.

Concrete sample compression strength after 7 and 28 days of hardening were determined (Figure 12).

It is seen that by raising the waste amount in the mixture, concrete compression strength lowers, for the concrete sample CW1, compression strength was 91%, counting from the sample compression strength of CW0. While investigating the compression strength change, all aggregates were changed with concrete waste. It can be seen that by using waste, the compression strength fell by 18%, while compared with the sample compression strength when there is no concrete waste. A decrease in strength can be explained by a weakened connection between the aggregate from waste and newly formed cement rock.

Using experiments and calculations, the formed sample resistance to frost was determined. Sample destruction when freezing and thawing was determined with experiments, while the predicted exploitation resistance to frost was determined with calculations. Predicted constructional concrete resistance to frost can be determined according to physical and structural characteristics [26]. According to scientists’ recommendations and used methodologies [24,25] with experiments, concrete sample structural characteristics (Table 5) were determined and the predicted exploitation sample resistance to frost was calculated.

According to the research results (Table 5), it can be seen that all concrete sample characteristics—general porosity—is similar. The authors [29] state that general sample porosity 90% depends on water amount used in mixing, which in these investigated mixtures were the same.

Predicted sample exploitation resistance to frost was calculated according to sample structural characteristics by the fixating sample destruction start. According to these indicators, it can be seen that the highest predicted exploitation resistance to frost is of the concrete samples, which are prepared by using natural aggregates, while using crushed waste this size was smaller.

Effective sample porosity size shows how many pores and empty spaces the sample has that a chance to be filled with water during the the 3 day submerging process have. General sample porosity shows how many pores and empty spaces have a chance to be filled with water throughout a very long period (for example, when the samples are used in building facade, where they would be constantly dried and from time to time would freeze and thaw). According to Table 5 data it can be seen, that concrete with waste more easily fill with water after 3 day period and has lower porous space reserve values. Porous space reserve also reflects size, how many open pores and empty spaces the material has, which are like a reserve, filled through long-term period.

From Table 5, it can be seen that as the predicted resistance to frost decreases, the waste amount in the mixture increases.

**Figure 13:**
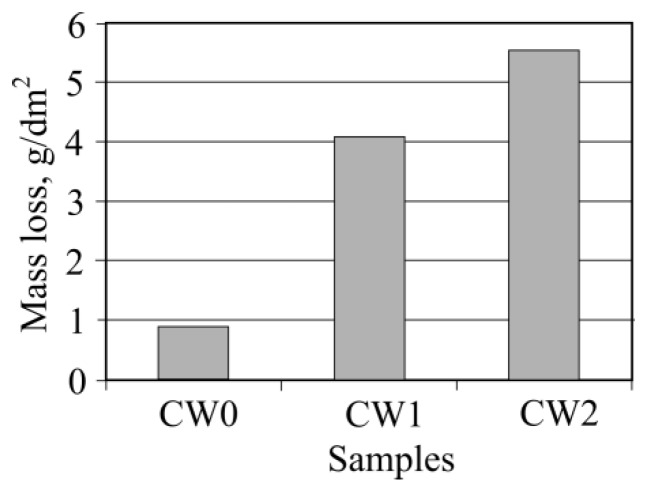
Dependence of samples’ mass losses after 56 test cycles on the amount of concrete waste.

To effectively investigate concrete resistance to frost, it is necessary to determine not only structural concrete characteristics and to calculate the predicted exploitation resistance to frost, but to also carry out their comparison analysis using the resistance to frost values obtained during the experiment process. The determined sample resistance to frost results are shown in Figure 13. Figure 13 shows that no samples reached the maximum allowed mass loss of 1.5 kg/m^2^ (15 g/dm^2^). The best results, according to this indicator, were obtained during the experiments of sample CW0, which used natural aggregates. Its mass loss after 56 freezing and thawing cycles was only 0.87 g/dm^2^. The sample surface had no visible big cracks; the contact zone between the cement rock and aggregate was undamaged. On some parts of the cement rock, only insignificant micro-cracks were visible.

**Figure 14:**
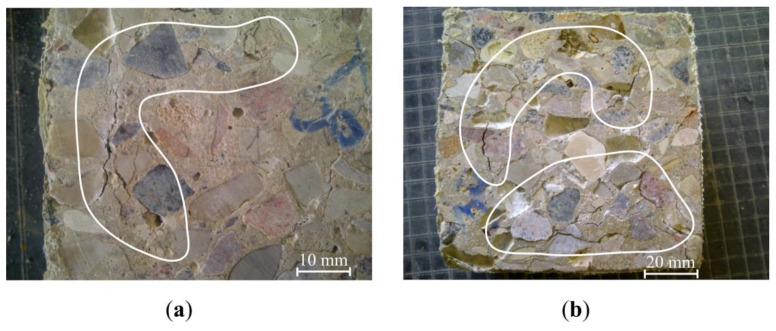
View of the samples after 56 frost resistance testing cycles, (**a**) CW1 sample; (**b**) CW2 sample.

In the concrete samples, where part of the coarse aggregate was replaced by waste (CW1), and samples where used aggregates were only made of waste (CW2), mass loss was highest, however, they did not reach maximum values. In these samples, after 56 cycles, significant cracks appeared on the sample surface and contact zone between the aggregate and cement rock, which are marked in Figure 14a,b.

The appearance of cracks in the contact zone betweenthe aggregate and cement rock can be explained by a weakened connection between the aggregate from waste and newly formed cement rock.

After comparing the results shown in Table 5, which presents the predicted exploitation sample resistance to frost, and the results shown in Figure 14, it can be seen that the prediction method partially reflects the obtained results through the experiments.

The investigated concrete costs comparison is shown in Table 6. The presented concrete mixture costs are valid for the Lithuanian building construction market and were obtained from a concrete producing company at the time of writing this article. The concrete with crushed waste landfill dumping charge is calculated according to subsideries, which the concrete mixture manufacturer could get from the government when using waste materials.

After researching how concrete properties change when using crushed concrete waste instead of coarse natural aggregate for mixing, it can be determined what concrete products—as in, what concrete class products—could be produced. From the research results, it is clear that by changing the natural aggregate to concrete waste after hardening lowers the mechanical characteristics of the obtained concrete. However, not all concrete products can be produced from higher class concrete, similar to the fact that not all products need to have a high resistance to frost. For products which do not have a high resistance to frost and mechanical characteristic requirements, crushed concrete waste can be used perfectly fine. In this way, a high amount of waste can be used up.

## 4. Conclusions

By using the standard research method to determine the main aggregate characteristics, it is evident that coarse aggregate obtained from concrete waste differs from aggregate made with natural rock. It was also noted that the concrete waste cleavage indicator and absorption can be significantly higher than natural aggregate.

The research conducted using concrete samples, for which crushed concrete waste (4/16) was used as coarse aggregates, indicates that the properties of hardened concrete depend on the amount of concrete used in fresh concrete. The concrete density and compression strength decreased (up to 8% and up to 18%, respectively), and absorption increases (almost twice) due to the aggregates derived from crushed concrete waste, since the cleavage indicator is two times higher, while water absorption is four times higher than in natural aggregate.

Using a raster electron microscope to observe aggregates from crushed concrete waste and the contact zone of newly hardened cement rock, demonstrates that coarse aggregate from waste does not bond well to the newly formed cement rock, which impairs the mechanical property of concrete and causes a deterioration in its physical properties.

Concrete includes coarse (4/16 mm) aggregates obtained from crushed concrete waste, resulting in a lower compression strength of 5–8%, and a lower density—8–18%. Notably, there is almost a 200% rise in the absorption rates, since the concrete structure and the use of fractional concrete waste properties change when compared with natural aggregates.

Even though effective and general sample porosity increased after adding concrete waste, porous space reserve in these samples became smaller than 30%. This indicator is a strong influence when predicting the resistance to frost. Due to this, and after calculating all structural parameter influences, we determined that the predicted exploitation resistance to frost declined by 19%–28%.

After measuring the concrete sample resistance to frost with an experimental method, it was determined that the mass loss after 56 freezing and thawing cycles amounted to 0.87 g/dm^2^ when investigating concrete samples with no concrete waste in their composition. By adding part of the concrete waste (instead of natural aggregates), the mass loss rose 4.7 times, whereas the mass loss rose 6.2 times when coarse concrete aggregates were replaced by crushed waste. 

## Figures and Tables

**Table 1:** Main coarse aggregate characteristics.

Coarse Aggregate	Bulk Density, g/cm^3^	Particle Density, g/cm^3^	Emptiness, %	Water Absorption After 48 h, %	Cleavage Indicator, %
Reference	[21]	[22]	[21]	[22]	[23]
Gravel	1.43	2.60	42	1.80	14
Crushed waste	1.25	2.30	46	7.80	34

**Table 2:** Concrete compositions with various amounts of crushed concrete waste for 1 m^3^ of mixture.

Fresh Concrete	Cement	Crushed Concrete Waste	Gravel	Sand	Water	W/C
CW0	465	-	1360	376	200	0.43
CW1	465	560	800	376	200	0.43
CW2	465	1360	-	376	200	0.43

**Table 3:** Results of exothermic effects.

Fresh Concrete Marking	Exothermic Effect Highest Temperature (T_max_), °C	Time when Highest Temperature was Achieved
CW0	31.28	14 h 28 min
CW1	31.24	14 h 59 min
CW2	30.50	15 h 34 min

**Table 4:** Areas of complex phases noticed in the cross-section of concrete samples, %.

Noticed Zone Color and Name	CW0	CW1	CW2
White color (pores)	0.64	0.81	1.43
Light gray color (newly formed cement rock with fine aggregate)	44.83	45.21	46.63
Dark gray colour and black color (cement rock from concrete waste and natural aggregates from concrete waste)	-	53.98	50.95
Black color (coarse natural aggregates)	54.54	-	-

**Table 5:** Structural characteristics and predicted exploitation frost resistance of the samples.

Parameter	CW0	CW1	CW2
Effective sample porosity, % [25]	7.94	9.27	10.25
General sample porosity, % [25]	13.81	14.08	14.61
Porous space reserve, % [25]	42.48	34.13	29.84
Conditional pore and capillary wall thickness [25]	6.24	6.11	5.84
Structural direction unevenness indicator [25]	5.00	3.50	3.70
Capillary mass stream speed in a vacuum in the direction of freezing [25]	1.05	1.04	0.80
Capillary mass stream speed in a vacuum perpendicular to freezing direction [25]	1.65	1.60	1.26
Capillary mass stream speed in normal conditions [25]	0.36	0.38	0.40
Predicted exploitation resistance to frost, in cycles [26]	241	195	172

**Table 6:** Concrete mixture costs and landfill dumping charge comparison.

Concrete Mixture	CW0	CW1	CW2
Cost, Eur/m^3^	85.91	77.41	62.79
Landfill dumping charge, Eur per ton	52	47	38
